# The secreted Ly6/uPAR-related protein-1 suppresses neutrophil binding, chemotaxis, and transmigration through human umbilical vein endothelial cells

**DOI:** 10.1038/s41598-019-42437-x

**Published:** 2019-04-11

**Authors:** Sudha Swamynathan, Anil Tiwari, Chelsea L. Loughner, John Gnalian, Nicholas Alexander, Vishal Jhanji, Shivalingappa K. Swamynathan

**Affiliations:** 1 Department of Ophthalmology, University of Pittsburgh School of Medicine, Pittsburgh, USA; 2 School of Biological Sciences, University of Pittsburgh, Pittsburgh, USA; 3 Department of Cell Biology, University of Pittsburgh School of Medicine, Pittsburgh, USA; 4 Fox Center for Vision Restoration, University of Pittsburgh School of Medicine, Pittsburgh, USA; 5 McGowan Institute of Regenerative Medicine, University of Pittsburgh, Pittsburgh, USA; 6 Present Address: Lake Erie College of Osteopathic Medicine, Greensburg, PA USA

## Abstract

The secreted Ly-6/uPAR Related Protein-1 (SLURP1) is an immunomodulatory protein that promotes corneal immune- and angiogenic-privilege. Here, we have examined the influence of SLURP1 on neutrophil-vascular endothelial cell interactions using human umbilical vein endothelial cells (HUVEC) and differentiated neutrophil-like HL-60 (dHL-60) cells, or primary human neutrophils. SLURP1 blocked the tumor necrosis factor-alpha (TNF-α)-activated dHL-60 cells (*i*) binding to TNF-α-activated HUVEC with a concurrent reduction in endothelial cell adhesion molecule E-selectin, (*ii*) transmigration through TNF-α-activated confluent HUVEC monolayer by stabilizing VE-cadherin and β-catenin on endothelial cell cytoplasmic membranes, (*iii*) chemotaxis towards chemoattractant formyl Met-Leu-Phe (fMLP) coupled with their decreased polarization, and (*iv*) TNF-α-stimulated matrix metalloproteinase-9 (MMP9) expression and activity. SLURP1 also suppressed the primary human neutrophil chemotaxis, and interaction with HUVEC. Furthermore, SLURP1 suppressed fMLP-induced phosphorylation of protein kinase-B (AKT) in dHL-60 cells. Collectively, these results provide evidence that SLURP1 suppresses neutrophil (*i*) docking on HUVEC cells by decreasing endothelial cell adhesion molecule E-Selectin production, (*ii*) transmigration through HUVEC monolayer by stabilizing endothelial cell membrane localization of VE-cadherin and β-catenin complex and promoting their barrier function, and (*iii*) chemotaxis by modulating their polarization and TNF-α-stimulated MMP9 production.

## Introduction

The cornea is an immune- and angiogenic-privileged tissue that elicits inflammatory angiogenic response only to acute, but not mild insults. Disrupted immune-privilege results in unregulated inflammation and associated corneal neovascularization (CNV), a predominant reason for cornea-related visual impairment in the world^1^. Corneal immune- and angiogenic-privilege is regulated by a diverse set of protective molecules that block inflammatory and angiogenic response to mild insults, and are rapidly down-regulated during acute infections and injury, allowing protective inflammation to develop^1–9^. Corneal angiogenic- and immune- privilege is governed by the balance between pro-inflammatory factors such as tumor necrosis factor alpha (TNF-α), vascular endothelial growth factor-C (VEGF-C), VEGF-A, and anti-inflammatory factors like angiostatin, endostatin, soluble VEGF Receptor-3 (VEGF-R3), VEGF-R1, pigment epithelium derived factor (PEDF), thrombospondin-1 (TSP1), TSP2 and programmed death ligand-1 (PD-L1)^1,3,7,10–12^. When this balance tilts in favor of pro-angiogenic factors, heme- and lymph-angiogenesis occurs, resulting in corneal edema and opacity. Previously, we demonstrated that the secreted Ly6/uPAR-related protein-1 (SLURP1) contributes to corneal immune- and angiogenic-privilege in healthy corneas where it is abundantly expressed, and is sharply downregulated in response to acute pro-inflammatory signals, facilitating progression of beneficial inflammation when needed^13,14^.

SLURP1 is an 88 amino acids peptide secreted into the tear film, saliva, sweat, urine and plasma, and belongs to the family of leukocyte antigen-6 (Ly6) proteins^15^. SLURP1 is expressed relatively abundantly in the cornea, moderately in the skin, oral, bronchial, and other mucosal epithelia^16–18^, and at a low level in various cells of the immune system and the sensory nervous system^19–23^. SLURP1 is considered a late marker of skin keratinocyte differentiation^24^. NMR analysis revealed conformational heterogeneity of SLURP1 in aqueous solution, associated with cis-trans isomerization of the Tyr39-Pro40 peptide bond^25^. SLURP1 stimulates acetylcholine synthesis by serving as a selective allosteric antagonist of α7-nAchR and facilitates functional development of T cells^23,26,27^. SLURP1 suppresses TNF-α production by T-cells, IL-1 β and IL-6 secretion by macrophages, IFN-γ-induced upregulation of ICAM-1, and IL-8 secretion by human intestinal enterocytes^28^. Deletions or mutations in human *SLURP1* gene cause inflammatory autosomal recessive palmoplantar keratoderma called Mal-de-Meleda^29–31^. *Slurp1*-null mice display hyperproliferation of keratinocytes and palmoplantar keratoderma with accumulation of lipid granules in the stratum corneum, mimicking Mal-de-Meleda^32^. A survey of the available gene expression datasets revealed that Slurp1 is downregulated in pro-inflammatory conditions^33–36^. Taken together with the role of SLURP1 in Mal de Meleda, these changes suggested an important role for SLURP1 in modulating inflammation. Consistent with this, we previously demonstrated that Slurp1 serves as an immunomodulatory molecule by scavenging extracellular urokinase-type plasminogen activator (uPA)^13,18,37^, and blocks human umbilical vein endothelial cell (HUVEC) angiogenesis *in vitro* by suppressing TNF-α-activated nuclear translocation of NFκB^14^.

In this report, we evaluate the influence of SLURP1 on endothelial cell-neutrophil interaction using HUVEC and differentiated neutrophil-like dHL-60 cells as well as primary human neutrophils, and present evidence that SLURP1 (*i*) suppresses binding of dHL-60 cells and primary human neutrophils to HUVEC, and dHL-60 polarization and chemotaxis towards chemoattractant formyl-Met-Leu-Phe (fMLP) by modulating PI3K/AKT pathway, (*ii*) impedes with dHL-60 transmigration through HUVEC monolayer by promoting membrane localization of cell adhesion molecules VE-cadherin and β-catenin and stabilizing the HUVEC barrier function.

## Results

### SLURP1 impedes with neutrophil binding to HUVEC

**Figure 1:**
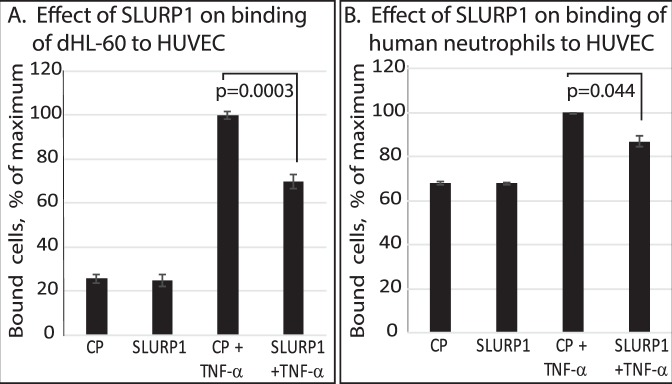
SLURP1 suppresses the interaction of TNF-α-activated dHL-60 and primary human neutrophils with TNF-α-activated HUVEC monolayer. The number of unstimulated or TNF-α-activated (**A**) dHL-60 cells and (**B**) primary human neutrophils bound to a confluent HUVEC monolayer upon treatment with control protein (CP; mock purified control protein from the parental strain without SLURP1 expression vector) or SLURP1 is shown. An unpaired t test was used to compare means of CP + TNF-α and SLURP1 + TNF-α and p value is shown in the graph. The data presented is representative of three independent experiments, each with a minimum of four replicates.

As (*i*) SLURP1 impedes with neutrophil influx into healthy corneas^13,18,37^, and (*ii*) neutrophil binding to endothelial cell walls is an essential prerequisite for their extravasation from post-capillary venules^38–40^, we evaluated the influence of SLURP1 on neutrophil-endothelial interaction by quantifying the binding of TNF-α-activated dHL-60 and primary human neutrophils to TNF-α-activated HUVEC monolayer. The number of dHL-60 cells and primary neutrophils bound to HUVEC monolayer increased significantly with TNF-α (Fig. 1). In contrast, treatment with SLURP1 resulted in a 30 and 13% decrease in binding of dHL-60 cells and primary human neutrophils, respectively, to TNF-α-activated HUVEC (Fig. 1), revealing that SLURP1 impedes with neutrophil binding to HUVEC.

### SLURP1 regulates the expression of E-Selectin on HUVEC surface

**Figure 2:**
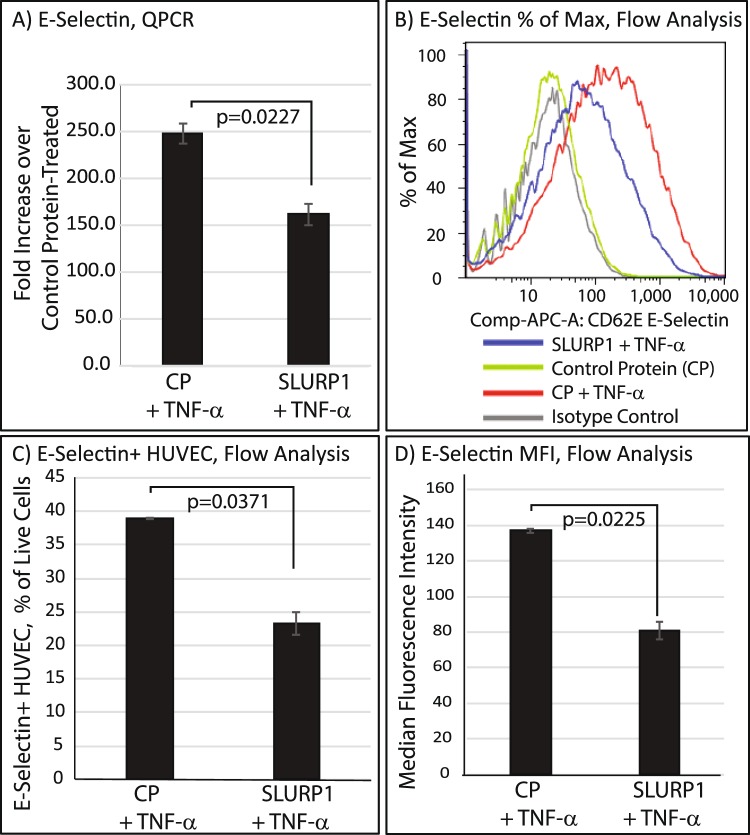
SLURP1 suppresses the expression of E-Selectin on HUVEC surface. (**A**) Comparison of *E-Selectin* transcript levels in TNF-α-activated HUVEC treated with control protein (CP; mock purified control protein from the parental strain without SLURP1 expression vector) or SLURP1 by QPCR. The data presented is an average of two experiments, each with three replicates. (**B**–**D**) Flow cytometry showing percentage of E-Selectin-positive cells and their median fluorescence intensity. In each experiment, 30,000 cells were analyzed. The data presented is representative of three independent experiments with at least two replicates in each. An unpaired t test was used to compare the values obtained with CP + TNF-α and SLURP1 + TNF-α treated HUVEC.

To determine the mechanistic basis for decreased dHL-60 binding to TNF-α-activated HUVEC in the presence of SLURP1, we next examined the effect of SLURP1 on E-selectin, a cell adhesion molecule highly expressed on endothelial cell surface that plays an important role in neutrophil-endothelial cell interaction^38–40^. QPCR revealed a robust 250-fold increase in E-selectin transcripts in HUVEC 3 h post-TNF-α-activation, which was significantly suppressed by addition of SLURP1 (Fig. 2). Consistent with these results, flow cytometry revealed a significant increase in the number of E-Selectin-positive HUVEC and their median fluorescence intensity (MFI) upon TNF-α-activation, which was significantly decreased upon treatment with SLURP1 (Fig. 2). Collectively, these results suggest that SLURP1 suppresses neutrophil binding to endothelial cells (Fig. 1) by suppressing the expression of E-selectin on TNF-α-activated HUVEC surface (Fig. 2).

### SLURP1 suppresses neutrophil transmigration and chemotaxis

**Figure 3:**
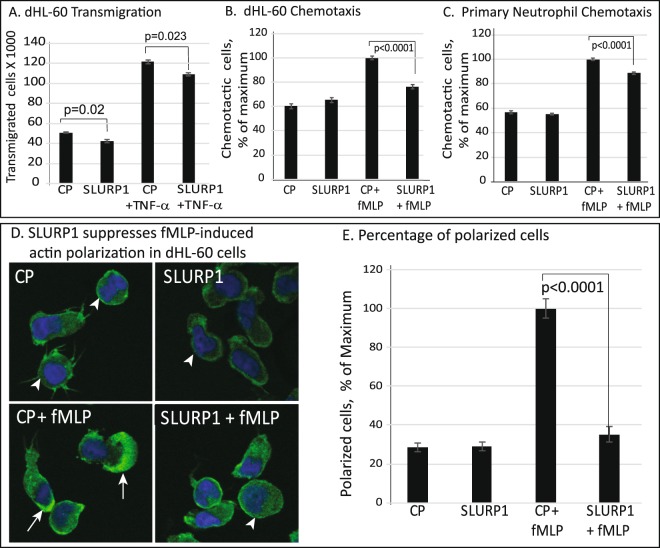
SLURP1 suppresses transmigration of TNF-α-activated dHL-60 through TNF-α-activated HUVEC monolayer, and neutrophil chemotaxis. (**A**) Number of TNF-α-activated dHL-60 cells transmigrated towards fMLP through a TNF-α-stimulated confluent HUVEC monolayer. The data shown is an average of four independent experiments, each with three replicates. (**B**,**C**) The number of (**B**) dHL-60 cells and (**C**) primary neutrophils migrated towards fMLP under different conditions tested is shown, quantified using a standard curve. The data shown is an average of three independent experiments, each with three replicates. (**D**) Phalloidin staining to visualize actin polymerization in dHL60 cells exposed to different conditions. Polarized cells are marked by arrows and those that are not polarized are indicated by arrowheads. (**E**) Percent of polarized dHL-60 cells under different conditions tested was manually counted. The data presented is the average of three independent experiments, with the polarized cells counted in three different microscopic fields in each experiment. CP, control protein mock purified from the parental strain without SLURP1 expression vector; fMLP, formyl Met-Leu-Phe tripeptide chemoattractant.

As neutrophil transmigration through endothelial barrier is essential for neutrophil recruitment to the site of injury^38–40^, we next examined the effect of SLURP1 on transmigration of dHL-60 cells through HUVEC monolayer, with fMLP as a chemoattractant. The number of dHL-60 cells that transmigrated through a confluent HUVEC monolayer increased significantly with TNF-α treatment (Fig. 3A). Treatment with SLURP1 resulted in a statistically significant 11% decrease in dHL-60 transmigration through TNF-α-activated HUVEC (Fig. 3A).

Next, we quantified the effect of SLURP1 on chemotaxis of dHL-60 and primary human neutrophils towards chemoattractant fMLP using Boyden chambers. While the CP-treated dHL-60 cells responded well to fMLP as evidenced by the increased number of migrated cells in the lower chamber, SLURP1-treated dHL-60 cells failed to do so (Fig. 3B). Consistent with these results, primary human neutrophils also displayed a good chemotactic response to fMLP, which was significantly decreased upon SLURP1 treatment (Fig. 3C).

Considering that neutrophil polarization is essential for their chemotaxis to the site of injury, we next examined the effect of SLURP1 on dHL-60 cell polarization by staining the actin cytoskeleton with phalloidin. dHL-60 cells were exposed to a uniform concentration of 100 nm fMLP for 20 min and stained with phalloidin. Consistent with the decreased transmigration and chemotaxis in the presence of SLURP1 (Fig. 3A–C), treatment of dHL-60 cells with SLURP1 significantly decreased the fraction of polarized cells quantified by actin polymerization (Fig. 3D,E). Together, these results demonstrate that SLURP1-mediated suppression of dHL-60 transmigration and chemotaxis is accompanied by their diminished polarization.

### SLURP1 stabilizes endothelial cell junctions

**Figure 4:**
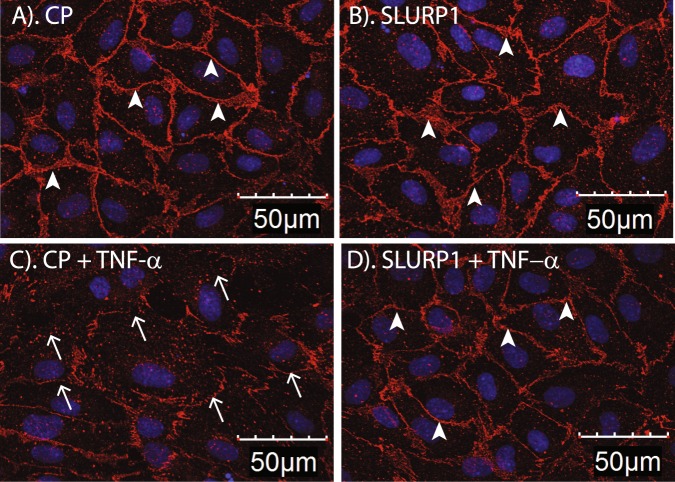
SLURP1 promotes VE-Cadherin presence in HUVEC cell junction. Immunofluorescent stain reveals abundant presence of VE-Cadherin (red; indicated by arrowheads) at junctions of confluent HUVEC treated with control protein (CP, control protein mock purified from the parental strain without SLURP1 expression vector) (**A**), or SLURP1 (**B**). Activation by TNF-α disrupted VE-Cadherin at HUVEC junctions (open arrows; **C**), which was restored by treatment with SLURP1 (arrowheads; **D**). Nuclei are counterstained with DAPI (blue). The data presented is representative of three independent experiments.

Adherens junctions containing VE-cadherin enhance the integrity of endothelial cell junctions and suppress the permeability of the vascular endothelium^38–40^. To determine whether SLURP1 prevents TNF-α-mediated destabilization of endothelial cell junctions, we examined the expression of VE-cadherin in cell junctions in HUVEC exposed to TNF-α in the presence of CP or SLURP1. Immunofluorescent staining revealed abundant VE-cadherin expression at CP- or SLURP1-treated HUVEC cell junctions (Fig. 4A,B), which was disrupted in TNF-α-activated HUVEC junctions (Fig. 4C) but was maintained at close to normal levels in the presence of SLURP1 (Fig. 4D).

**Figure 5:**
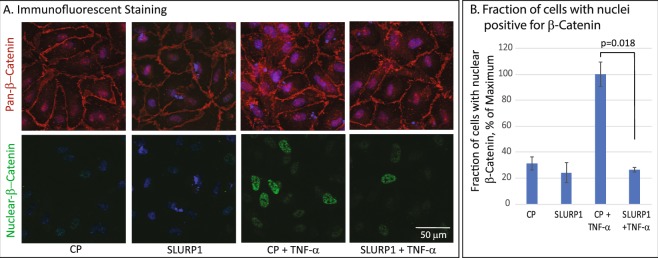
SLURP1 suppresses nuclear localization of β-catenin in TNF-α-activated confluent HUVEC monolayer. (**A**) Immunofluorescent stain with anti-pan β-catenin (upper panels; red), or anti-nuclear β-catenin antibody (lower panels; green) is shown. (**B**) Fraction of HUVEC with nuclear localization of β-catenin under different conditions tested. Cells with nuclear β-catenin counted by using a program in Metamorph. The data presented is representative of three independent experiments. CP, control protein mock purified from the parental strain lacking SLURP1 expression vector.

Considering that the stable association of VE-cadherin with β-catenin is essential for endothelial cell membrane integrity, and that pro-inflammatory cytokines disrupt this interaction, we next evaluated the expression and localization of β-catenin in TNF-α-activated HUVEC. Immunofluorescent stain with an antibody that recognizes all forms of β-catenin revealed destabilization of the membrane-bound β-catenin coupled with increased nuclear β-catenin upon HUVEC activation by TNF-α, which was restored close to normal levels by treatment with SLURP1 (Fig. 5). Consistent with these results, use of an antibody that recognizes only the nuclear β-catenin revealed increased frequency of nuclear β-catenin expression upon activation by TNF-α, which was reversed upon treatment with SLURP1 (Fig. 5). Collectively, these results demonstrate that SLURP1 stabilizes endothelial cell junctions by maintaining normal level of VE-cadherin (Fig. 4) which in turn retains β-catenin at the cell junctions (Fig. 5).

### SLURP1 downregulates matrix metalloproteinase-9 (MMP9) expression in dHL-60 cells

**Figure 6:**
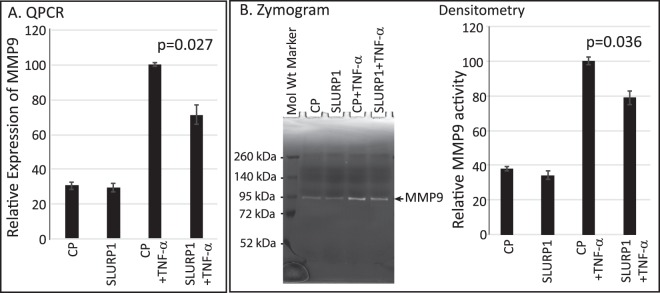
SLURP1 suppresses MMP9 expression and activity. (**A**) QPCR evaluation of MMP9 transcript levels in TNF-α-activated dHL-60 cells in the presence or absence of SLURP1. (**B**) Representative zymogram showing MMP9 activity in cell culture supernatants under different conditions tested. Relative MMP9 activity under these conditions, quantified by densitometry is shown on the right. The data presented is an average of three independent experiments, each with a minimum of two replicates. CP, control protein mock purified from the parental strain lacking SLURP1 expression vector.

As neutrophil-secreted MMP9 facilitates their migration, we tested if SLURP1 mitigates TNF-α-induced MMP9 production in dHL-60 cells. QPCR revealed that TNF-α exposure upregulated MMP9 transcript levels by 3.3-fold, which was significantly decreased to 2.3-fold upon SLURP1 treatment (Fig. 6A). Consistent with these results, gelatin zymography revealed increased MMP9 protease activity upon TNF-α activation that was significantly decreased in the presence of SLURP1 (Fig. 6B).

### SLURP1 suppresses fMLP-induced AKT phosphorylation

**Figure 7:**
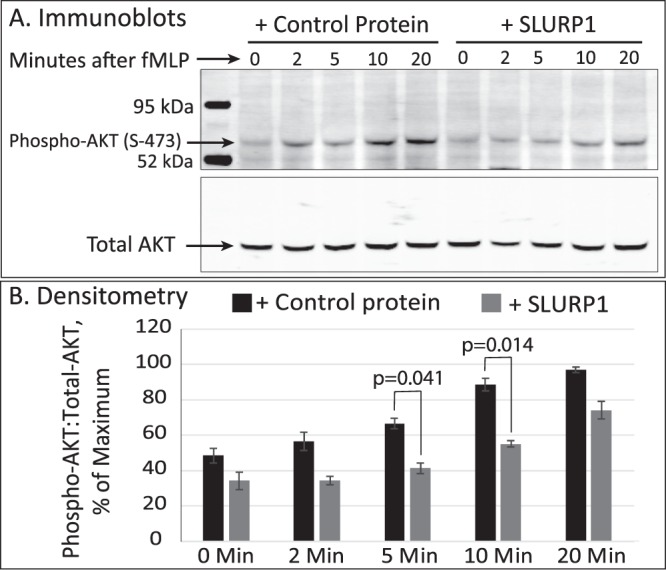
SLURP1 suppresses fMLP-induced AKT phosphorylation. (**A**) Lysate from dHL-60 cells preincubated with control protein (CP, mock purified control protein from the parental strain lacking SLURP1 expression vector) or SLURP1 and exposed to 1 μM fMLP (formyl Met-Leu-Phe tripeptide chemoattractant) for 2 to 20 minutes was probed with anti-phospho-AKT (S-473) & anti-total AKT antibodies. Full length, uncropped blots can be seen in Supplemental Fig. 3. (**B**) The ratio of phospho-AKT: total-AKT quantified by densitometry is shown. The data presented is an average of three independent experiments. An unpaired t test was used to compare phospho-AKT: total-AKT ratios in CP- and SLURP1-treated dHL-60 cells at each time point.

Considering that the PI3K/AKT pathway plays a key role in angiogenesis and inflammation by regulating NFκB activity^41^ and also is a major pathway linking fMLP to actin reorganization in neutrophils^42,43^, we tested if SLURP1 functions through PI3K/AKT pathway by evaluating fMLP-stimulated AKT activation in dHL-60 cells. The lysate from CP- or SLURP1-treated dHL-60 cells exposed to fMLP was simultaneously probed with anti-total AKT and anti-phospho-AKT (S-473) antibodies (Fig. 7). The phospho-AKT: total AKT ratio quantified by densitometry revealed that fMLP-stimulated dHL-60 cells displayed elevated AKT phosphorylation in the presence of CP, but not SLURP1 (Fig. 7), consistent with the possibility that SLURP1 modulates neutrophil interaction with HUVEC by inhibiting AKT phosphorylation.

## Discussion

**Figure 8:**
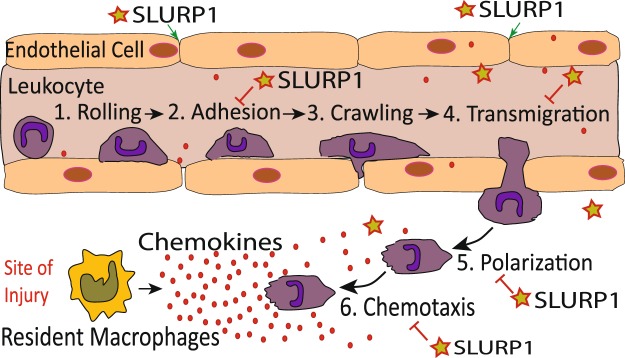
Schematic summary of the effects of SLURP1 on neutrophil-endothelial interaction. Data presented in this report demonstrate that SLURP1 acts as an immunomodulatory protein by stabilizing the endothelial barrier function (green arrows), and by suppressing neutrophil (*i*) binding to endothelial cell surface, (*ii*) transmigration, (*iii*) polarization and (*iv*) chemotaxis (red blunt-ended lines).

Previously, we demonstrated that SLURP1, an immunomodulatory molecule abundantly expressed in healthy corneas, inhibits HUVEC tube formation by suppressing NFκB^13,14,18,37^. Here, we provide evidence demonstrating that SLURP1 inhibits the progression of inflammation by influencing both neutrophils and endothelial cells at multiple steps, including suppression of neutrophil (*i*) docking on HUVEC cells by decreasing the production of endothelial cell adhesion molecule E-Selectin, (*ii*) transmigration through HUVEC monolayer by stabilizing endothelial cell membrane localization of VE-cadherin and β-catenin complex and promoting their barrier function, and (*iii*) chemotaxis by modulating their polarization and TNF-α-stimulated MMP9 production (Fig. 8). We also provide evidence that SLURP1 suppresses fMLP-stimulated AKT phosphorylation in dHL-60 cells, raising the possibility that SLURP1 suppresses neutrophil extravasation and chemotaxis by interfering with the PI3K/AKT pathway.

Neutrophils can detect shallow gradients of chemoattractants released in response to injury and respond by rapid extravasation and chemotactic migration. Tethering and rolling, among the first steps in the process of neutrophil extravasation, depend on efficient interaction between neutrophil surface glycoproteins and endothelial selectins^38–40^. Upon activation by pro-inflammatory cytokines, endothelial cells express intercellular adhesion molecule-1 (ICAM1 or CD54) vascular cell adhesion molecule-1 (VCAM1 or CD106), E-selectin (CD62) and to a lesser extent, L-selectin (CD62L). The results presented here reveal that SLURP1 suppresses TNF-α-induced overexpression of E-selectin by HUVEC, resulting in decreased binding of dHL-60 cells. Previously, we reported that SLURP1 blocks TNF-α-induced NFκB nuclear translocation in HUVEC^14^. As TNF-α-induced upregulation of E-Selectin expression is mediated through NFκB^44^, it is likely that the observed SLURP1-mediated downregulation of E-Selectin (Fig. 2) is due to its inhibitory effect on nuclear translocation of NFκB^14^.

Following extravasation, neutrophil chemotaxis to the site of injury is an essential step in innate immune response. Neutrophil chemotaxis requires rapid and well-coordinated intracellular signaling to orchestrate cytoskeletal and membrane lipid reorganization that facilitates their migration towards higher concentration of the chemoattractant^45,46^. The data presented here reveal that SLURP1 impedes with fMLP-induced neutrophil polarization, and that SLURP1 decreases fMLP-induced AKT phosphorylation at Ser-473. These findings are consistent with the earlier reports that the AKT pathway influences F-actin formation during dHL-60 and neutrophil polarization^42,43^, and that AKT phosphorylation at Ser-473 is crucial for dHL-60 polarization and chemotaxis^42^.

VE-cadherin- and β-catenin-containing adherens junction complex plays an important role in maintaining a restrictive vascular endothelial barrier by enabling endothelial cells to adhere in a homophilic manner. During inflammation, transmigration of leukocytes through the endothelial lining is facilitated by transient re-distribution of VE-cadherin from cell-cell junctions, and the resulting movement of β-catenin to the nucleus^38–40^. The results presented here provide evidence that SLURP1 stabilizes the VE-cadherin-β-catenin complex, promoting endothelial barrier and restricting the transmigration of neutrophils. These results, taken together with the previous reports that (*i)* SLURP1 expression is downregulated in many metastatic tumors^47^, and (*ii*) the nuclear β-catenin levels are elevated in head and neck squamous cell carcinoma, basal cell carcinoma, prostate cancer, colorectal cancer and medulloblastoma^48,49^, suggest that SLURP1 serves as an anti-tumor agent by stabilizing β-catenin at cell membranes.

In summary, the current data demonstrate that SLURP1 suppresses neutrophil extravasation and chemotaxis by interfering with the PI3K/AKT pathway. Together with our previous reports^13,14,18,37^, this report establishes that SLURP1 suppresses unwarranted neutrophil influx by suppressing neutrophil (*i*) tethering on endothelial surface, (*ii*) transmigration through endothelial barrier (*iii*) polarization and chemotactic migration, and (*iv*) TNF-α-activated MMP9 production. Though the effect of SLURP1 on each of these steps in neutrophil recruitment is moderate, their cumulative effect is likely to be a significantly suppressed neutrophil influx into healthy mucosal tissues such as corneas where SLURP1 is abundantly expressed.

## Methods

All methods reported here were carried out in accordance with relevant guidelines and regulations of the University of Pittsburgh. All experimental protocols were reviewed and approved by the University of Pittsburgh Institutional Biosafety Committee (IBC; Protocol Number: IBC201600216). All experimental protocols involving human samples were reviewed and approved by the University of Pittsburgh Institutional Review Board (IRB; Protocol Number: PRO180070258).

### Cells and Reagents

Promyelocytic leukemia HL-60 cells (ATCC ref. no. CCL-240) were maintained in Iscove’s modified Dulbecco’s medium (IMDM) with 20% FBS and differentiated into neutrophil-like dHL-60 cells using 1.25% dimethyl sulfoxide (DMSO) in IMDM with 10% FBS for 6 days^50^. Efficiency of dHL-60 differentiation was confirmed by Grinwald-Giemsa staining (Supplemental Fig. 1). Peripheral blood neutrophils were isolated from blood of healthy volunteers collected following informed consent, using an IRB-approved method by MACSxpress Neutrophil Isolation Kit (Miltenyi Biotec, Bergisch Gladbach, Germany). The purity of isolated neutrophils was tested by flow cytometry for CD11b expression, and was routinely greater than 95%. Primary HUVEC pooled from different donors were grown in Endothelial Growth Medium-2 (EGM2) (Lonza, Portsmouth, NH). HUVECs were serum-starved in basal medium with 0.5% FBS for 2 to 6 h, and the experiments were carried out in the same medium. TNF-α was from Thermo Scientific (Pittsburgh, PA). Recombinant human 6X His-SLURP1 was expressed in yeast *Pichia pastoris* or *E. coli*, and partially purified using Ni-ion affinity column chromatography. The control protein (CP) used was mock purified in a similar manner from the parental strain lacking SLURP1 -expressing vector. In these assays, we used 1 μg/ml SLURP1, previously determined to be the lowest effective concentration in HUVEC tube formation and dHL-60 chemotaxis assays. See Supplemental Fig. 2 for the purity and identity of the partially purified SLURP1 and the CP.

### RNA Isolation, Reverse Transcription and Quantitative PCR

QPCR was employed to quantify the relative expression of MMP9 4 h after SLURP1- or CP-treated dHL-60 cells were treated with TNF-α (10 ng/ml). Mouse Moloney Leukemia Virus reverse transcriptase (Promega, Madison, WI) was employed for cDNA synthesis with 1 μg of total RNA isolated using EZ-10 mini-prep kit (Bio Basic Inc. Amherst, NY). QPCR assays were performed in duplicate with validated primers using GAPDH as endogenous control. For quantifying the influence of SLURP1 on E-selectin expression, HUVECs grown in complete medium were serum-starved overnight, preincubated with CP or SLURP1 for 30–60 min and treated with 10 ng/ml TNF-α for 3 hours. RNA extraction, cDNA synthesis and QPCR were performed as described above for dHL-60, using primers from realtimeprimers.com (E-selectin- forward: 5′-ATGCCTTTATG GCTGAAACC-3′; reverse: 5′-CCAAGATTTT ACAGCGAGCA-3′; MMP9- forward: 5′-CTCTGGAGGT TCGACGTG-3′; reverse: 5′-GTCCACCTGG TTCAACTCAC-3′).

### Flow Cytometry

The source, catalog numbers of different antibodies, and their dilutions used in this study is provided in Supplemental Table 1. Serum-starved HUVECs preincubated with CP or SLURP1 for one hour and activated by treating with 10 ng/ml TNF-α for 18 h were detached by accutase (B.D. Biosciences, Franklin Lakes, NJ) after washing once with PBS, washed in FACS buffer, blocked in 2% goat serum for 20 min, incubated with APC-conjugated primary antibody for E-Selectin (BioLegend, San Diego, CA) for one hour on ice, washed thrice with FACS buffer and 30,000 cells were analyzed using FACSAria cytometer with FlowJo Software.

### Immunofluorescent Staining

The source, catalog number of the antibodies and their dilutions used is provided in Supplemental Table 1. Confluent HUVECs grown on glass cover slips coated with vitronectin were serum-starved for 16 h in EBM2 supplemented with 0.5% FBS, pre-treated with CP or SLURP1 for 60 min and treated with TNF-α (10 ng/ml; two hours for VE-Cadherin staining, and four hours for β-catenin staining), fixed in 3% paraformaldehyde in PBS (20 min), washed thrice with PBS (5 min each), permeabilized with 0.1% Triton-X100 in PBS, washed thrice with PBS (5 min each), blocked with 10% goat-serum in PBS (1 h at 23 °C), incubated overnight with primary antibody (1:200 dilution in a humidified chamber at 4 °C), washed thrice with PBS (5 min each), incubated with Alexafluor-546-coupled second antibody (1:300 dilution) and Alexaflour-488 conjugated phalloidin (Molecular Probes, Carlsbad, CA) for 1 h at 23 °C, and washed thrice with PBS + 0.1% Tween-20 (PBST; 5 min each). Nuclei were stained with 4′,6-diamidino-2-phenylindole (DAPI) before mounting coverslips using Aqua-Poly/Mount (Polysciences, Warrington, PA). An Olympus IX81 confocal microscope (Center Valley, PA) was used for imaging after drying the slides overnight in dark and sealing the coverslips with clear nail polish. The number of cells with nuclear β-catenin was counted in a blinded manner using MetaMorph software (Molecular Devices, San Jose, CA).

### Determining cell polarization by staining F-actin with phalloidin

TNF-α-activated dHL-60 cells were washed and resuspended in IMDM + 1% BSA, pretreated for 30 min with SLURP1 or CP and exposed to the chemoattractant tripeptide fMLP for 20 min. Cells were fixed in buffered 3% paraformaldehyde, spun down on to slides using cytofunnels, air-dried, stained with Alexafluor-488 conjugated phalloidin and DAPI, washed and imaged as above. The number of polarized cells was counted in a blinded manner.

### dHL-60 or primary human neutrophils binding to HUVEC

Serum-starved confluent HUVEC monolayers in 96-well plates were pre-treated with CP or SLURP1, after which they were exposed to TNF-α (10 ng/ml) for 18 h. Calcein-labeled dHL-60 cells or neutrophils were incubated with CP or SLURP1 with or without TNF-α for 30 min or 15 min, respectively, and allowed to bind to similarly treated HUVECs for 30 min in IMDM with 1% BSA (migration medium) for dHL-60 and for 15 min in RPMI + 1% BSA for neutrophils. Unbound cells were removed by spinning the plate upside down for 5 min at 50 g. 100 μl of migration medium was added to the wells and the fluorescence read at 485/20 nm excitation and 528/20 nm emission, and the number of bound cells calculated using a standard curve.

### Chemotaxis and Transmigration

For chemotaxis, we used ChemoTx low volume 96-well plates with 5 μm pore size membrane (Neuroprobe, Gaithersburg, MD). 30 μl of migration medium with or without fMLP along with CP or SLURP1 was added to each well. dHL-60 and primary human neutrophils were incubated with CP or SLURP1 for 30 min (dHL-60) or 10 min (primary human neutrophils) and layered on the membrane and allowed to migrate for 90 min for dHL-60 and 45 min for neutrophils. Cells that migrated to the lower chamber were spun down, and quantified by measuring fluorescence and the cell number derived using a standard curve.

For transmigration, 60,000 HUVECs were seeded in 5 μm pore size Transwell inserts of a 24-well plate and grown to confluence for a minimum of three days. Serum-starved HUVECs were pre-treated with CP or SLURP1 for 30 min and activated with TNF-α for 18 h. dHL-60 cells were treated the same way as in binding experiment and allowed to transmigrate through similarly treated HUVECs for 4 h at 37 °C in a CO_2_ incubator. The cells that migrated into the lower chamber were collected and counted by flow cytometry.

### Immunoblots

dHL-60 cells were treated with CP or SLURP1 and exposed to TNF-α for 2 to 20 min, after which TNF-α-stimulation was stopped by adding 1 ml ice-cold PBS with 20 mM sodium fluoride. Cells were spun down and lysed in radioimmunoprecipitation assay (RIPA) buffer with protease- and phosphatase- inhibitors. Protein was quantified by BCA assay and equal amounts of lysate was separated on Bis-Tris gels, transferred to Immobilon-FL membrane (MilliporeSigma, Burlington, MA). Membrane was blocked and incubated overnight with primary antibodies and probed with IRDye secondary antibodies and visualized on Odyssey scanner (Li-Cor Biosciences, Lincoln, NE).

### Gelatin Zymography

dHL-60 cells preincubated in IMDM medium with 1% BSA with CP or SLURP1 were treated with 10 ng/ml TNF-α for 4 h. The culture supernatant was electrophoresed on a Nupage 10% gelatin gel, renatured, developed and stained following the protocol suggested by the manufacturer (Invitrogen, Carlsbad, CA).

## Supplementary information

Supplementary Information

## Data Availability

All data generated or analyzed during this study are included in this article and its Supplementary Information files.
