# Supplementary material for: The secreted Ly6/uPAR-related protein-1 suppresses neutrophil binding, chemotaxis, and transmigration through human umbilical vein endothelial cells
Source: Sci Rep. 2019 Apr 11;9:5898. doi: 10.1038/s41598-019-42437-x (PMC6459912; doi:10.1038/s41598-019-42437-x)
Supplement: Supplementary file 1 — Supplementary Information [file 41598_2019_42437_MOESM1_ESM.docx]

**The secreted Ly6/uPAR-related protein-1 suppresses neutrophil binding, chemotaxis, and transmigration through human umbilical vein endothelial cells**

**Sudha Swamynathan ^1^, Anil Tiwari ^1^, Chelsea L. Loughner ^1,#^, John Gnalian ^1,2^, Nicholas Alexander ^1^, Vishal Jhanji ^1^, and Shivalingappa K. Swamynathan ^1,3,4,5*^**

1) Department of Ophthalmology, University of Pittsburgh School of Medicine, Pittsburgh.

2) School of Biological Sciences, University of Pittsburgh, Pittsburgh.

3) Department of Cell Biology, University of Pittsburgh School of Medicine, Pittsburgh.

4) Fox Center for Vision Restoration, University of Pittsburgh School of Medicine, Pittsburgh.

5) McGowan Institute of Regenerative Medicine, University of Pittsburgh.

# Current Address: Lake Erie College of Osteopathic Medicine, Greensburg, PA.

*** Corresponding Author**: Shivalingappa K. Swamynathan, Ph.D.

University of Pittsburgh School of Medicine

203 Lothrop Street, Room 1025

Pittsburgh PA-15213.

Phone: 412-802-6437

Fax: 412-647-5880,

Email: [Swamynathansk@upmc.edu](mailto:Swamynathansk@upmc.edu)

**Running Title:** SLURP1 modulates neutrophil-endothelial interaction

**Keywords**: Cornea, Epithelium, SLURP1, Neutrophil, Endothelial cell

.

**Supplemental Information**


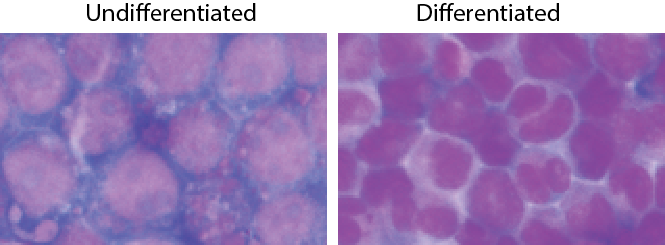


**Supplemental Figure 1**. Undifferentiated HL-60 cells compared with those differentiated to neutrophil like dHL-60 cells by treatment with 1.25 % DMSO for 6 days. These cells are stained with Grinwald-Giemsa stain to demonstrate the lobed nuclei in dHL-60 cells.

**
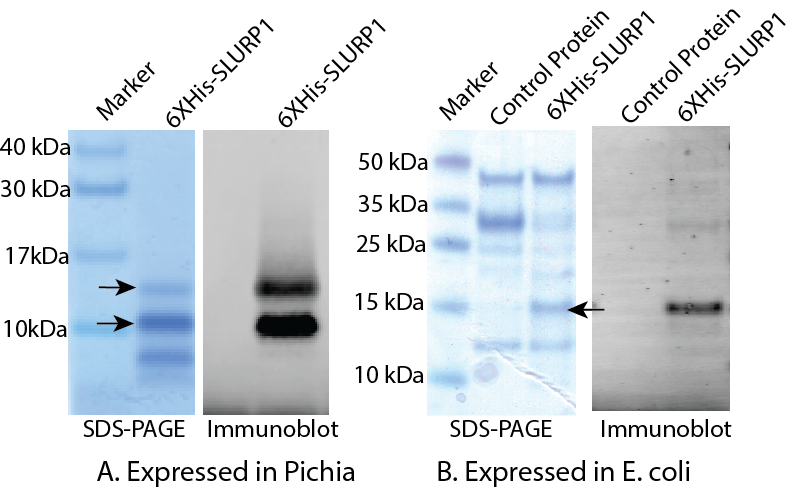
**

**Supplemental Figure 2**. Expression and partial purification of recombinant 6XHis-SLURP1 from A. Pichia pastoris and B. E. coli. Left panels show the SDS-PAGE profile, while the right panels show the corresponding immunoblot with anti-SLURP1 antibody. Mock-purified protein from the parental strains without SLURP1-expression vector was used as control protein (CP).


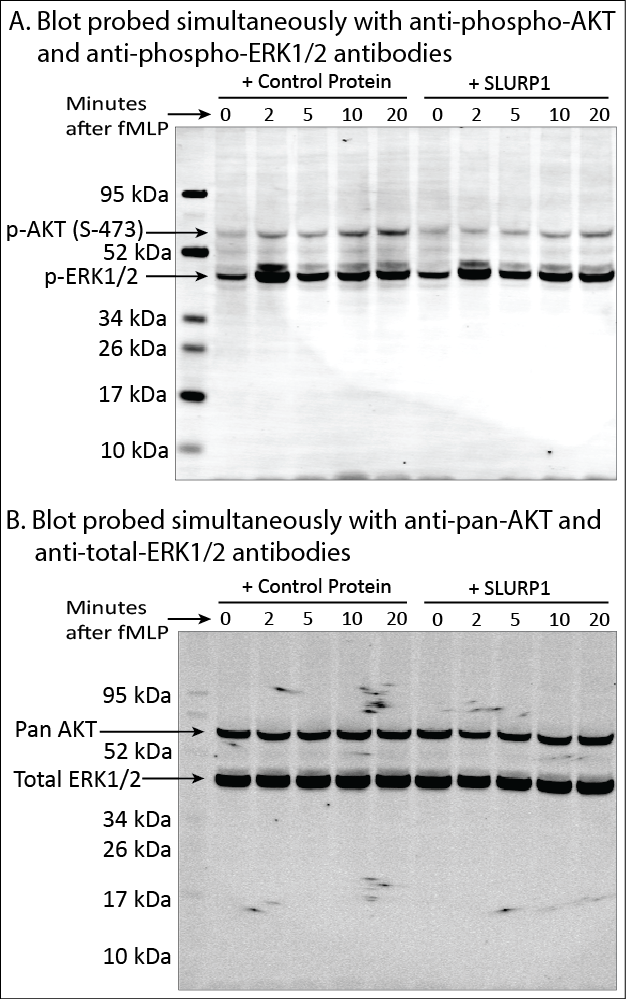


**Supplemental Figure 3**. **Uncropped images of the immunoblots used in figure 7A of the manuscript.** **A.** Blot probed simultaneously with anti-phospho-AKT and anti-phospho-ERK1/2 antibodies. Lysate from dHL-60 cells preincubated with control protein (CP) or SLURP1 and exposed to 1 μM fMLP for 2 to 20 minutes was probed with anti-phospho-AKT (S-473), and anti-phospho-ERK1/2 antibodies. **B.** The blot in panel A was simultaneously probed with anti-pan-AKT and anti-total-ERK1/2 antibodies. As densitometric quantitation revealed that SLURP1 has a significant suppressive effect on phosphorylation of AKT but not ERK1/2, only AKT is shown in the main manuscript. The blots shown are representative of three independent experiments.

**Supplemental Table 1**. List of antibodies, their source, catalog numbers, purpose and the dilutions used in the study. IF, Immunofluorescent stain; IB, Immunoblots; NA, Not Available.

|  | **Antibody** | **Source** | **Catalog Number** | **Purpose** | **Dilution Used** | **Final Concentration** |
| --- | --- | --- | --- | --- | --- | --- |
| 1 | Anti-VE-cadherin | Millipore | MABT134 | IF | 1:200. | 2.5 μg/ml |
| 2 | Anti-pan-β-catenin | Sigma | C2206 | IF | 1:500 | NA |
| 3 | Anti-Nuclear β-catenin | DSHB | PY489 | IF | 1:5 | 3 μg/ml |
| 4 | Anti-E-Selectin | BioLegend | 336011 | Flow | 1:35 | 2.8 μg/ml |
| 5 | Anti-SLURP1 | Santa Cruz Biotechnology | SC-98139 | IB | 1:200 | 1.0 μg/ml |
| 6 | Anti-CD11B | BioLegend | 301309 | Flow | 1:50 | 2.0 μg/ml |
| 7 | Anti-PanAKT | RND Systems | MAB2055 | IB | 1:2000 | 0.25 μg/ml |
| 8 | Anti-Phospho-AKT | Cell Signaling Technology | 4060P | IB | 1:2000 | NA |
| 9 | Anti-Actin | Santa Cruz Biotechnology | SC-1616 | IB | 1:600 | 0.2 μg/ml |
| 10 | Anti-total ERK1/2 | RND Systems | MAB1576 | IB | 1:500 | 1 μg/ml |
| 11 | Anti-Phospho-ERK1/2 | Cell Signaling Technology | 9101S | IB | 1:800 | NA |
